# Lifetime sexual violence and tobacco, alcohol, and cannabis use among French adults: A national survey

**DOI:** 10.1016/j.pmedr.2026.103547

**Published:** 2026-06-17

**Authors:** Noëlla Delmas, Anne Pasquereau, Jean-Baptiste Richard, Viêt Nguyen-Thanh, Fabienne El-Khoury Lesueur

**Affiliations:** a Sorbonne Université, INSERM, Pierre Louis Institute of Epidemiology and Public Health, IPLESP, Social Epidemiology, Mental Health and Addictions, ESSMA, F75012 Paris, France; b Santé publique France, Saint-Maurice, France

**Keywords:** Sexual violence, Tobacco, Alcohol, Cannabis, National survey

## Abstract

**Objective:**

Sexual violence can lead to significant health impacts, including higher risk of substance use. This study examines the association between lifetime experiences of sexual violence and substance use in a large nation-wide sample of French adults.

**Methods:**

Data were drawn from the 2017 Health Barometer, a cross-sectional survey of 25,319 French adults aged 18–75, conducted in Mainland France between January and July 2017. Five substance use outcomes were examined: daily tobacco use, heavy drinking, regular binge drinking, monthly cannabis use, and problematic cannabis use. Multivariable logistic regression models were used to assess the association between sexual violence and each of these outcomes.

**Results:**

A total of 9.5% of women and 2% of men reported having experienced sexual violence. In adjusted analyses, lifetime experience of sexual violence was associated with increased odds of all five substance use outcomes. Lifetime sexual violence was also linked to study outcomes in stratified analyses by sex, with particularly strong associations for monthly cannabis use (women aOR = 2.53, 1.90–3.34; men aOR = 2.10, 1.37–3.16).

**Conclusions:**

These findings support the need for integrated care addressing both sexual violence history and substance use among sexual violence victims-survivors.

## Highlights

• Lifetime sexual violence associated with higher odds of multiple substance outcomes.
• Sexual violence was linked to daily smoking, heavy drinking, and weekly binge drinking.
• Past-month cannabis use showed the largest association in both men and women.
• Both single and repeated sexual violence were associated with increased substance use risk.

## 1 Introduction

Sexual violence refers to any sexual act or attempt to obtain a sexual act through coercion, including rape or non-consensual touch (World Health Organization, 2002). Recent meta-analyses indicate that 8.7% of children globally have experienced contact sexual violence (Piolanti et al., 2025). Overall, the prevalence of sexual violence is higher among women than men, although data for the latter is limited (Borumandnia et al., 2020).

Sexual violence is a traumatizing experience associated with potentially lifelong mental health consequences. Among women, the experience of sexual violence increases the risk of harmful health behaviors, including high-risk sexual behaviors, eating disorders, and psychoactive substance use such as alcohol, cannabis, and tobacco (Fletcher, 2021). The use of these substances may suggest self-medication and a desire to regulate stress and face adversity (Mandavia et al., 2016). Having experienced sexual violence is also strongly associated with more frequent diagnoses of depression, suicidal ideation, anxiety and/or phobic disorders, and post-traumatic stress disorder (PTSD) (Hailes et al., 2019).

Although sexual violence is generally associated with higher use of tobacco, alcohol, and cannabis, the evidence base is constrained by small samples and a focus on specific populations (e.g., women, adolescents, military) and specific forms of sexual violence, particularly childhood sexual violence (Fletcher, 2021). Mixed-population findings also suggest that associations may vary by substance and sex (Choudhary et al., 2008; Draucker and Mazurczyk, 2013; Halpern et al., 2018).

To address these gaps, population-based data from a general adult sample are needed to better characterize the association between lifetime sexual violence and psychoactive substance use and to inform appropriate care and support for survivors.

Accordingly, this study investigates the association between lifetime experiences of sexual violence and the use of tobacco, alcohol, and cannabis in a national sample of French adults.

## 2 Methods

### 2.1 Study design and population

The present study drew data from the 2017 Health Barometer, a cross-sectional survey of the general population living in Mainland France carried out by the French National Public Health Agency (Santé publique France). Randomly-generated landline and mobile phone (dual frame; df) numbers lists were used to call participants up to 40 times using a computer-assisted telephone interviewing (CATI) system (Richard et al., 2017). For mobile phones, the selected interviewee was the person who picked up the phone. In the case of a landline call, a secondary randomization among eligible individuals, per the Kish method (Kish, 1949), made it possible to select the respondent person within the household. Thus, the sampling design was one-stage for cell interviews and two-stage for landline interviews.

Design weights, reflecting the individual selection probability, were calculated for the dual frame sample using information about the number of phone numbers generated, the number of phone numbers owned by the respondent (reported in the questionnaire), and the number of eligible persons in the household for landlines.

The Health Barometer survey was carried out from January 5th to July 18th 2017. The participation rate was 48.5% and the average interview duration was 31 min long. A representative sample of 25,319 French-speaking people aged 18 to 75 was drawn up.

Data from the survey were weighted by considering the probability of inclusion of an individual, and adjusted based on the structure of the population (age and sex stratification, education level, region of residence, household size, and urban area size), obtained from the 2016 employment survey from the French National Institute of Statistics and Economic Studies. The detailed survey protocol and its questionnaire are available online (Richard et al., 2018).

### 2.2 Measures

#### 2.2.1 Lifetime experiences of sexual violence

To determine whether respondents had experienced sexual violence, the following questions were asked: “Throughout your life, have you ever been forced to touch or be touched by someone in a sexual manner? Have you ever been forced into sexual activity against your will? Yes or No”. If answered yes, they were asked if it had happened several times, and their age at the time of the event (or at first experience if they've had several experiences).

#### 2.2.2 Psychoactive substance use outcomes

***Tobacco use outcome**.* One of the variables of interest in our study was the daily use of tobacco. We included data from respondents who declared themselves as smokers and answered yes to the question: “Do you smoke every day?”, or declared daily consumption of cigarettes (manufactured or roll-your-own). Exclusive e-cigarette users were excluded from the analysis, as they represent a heterogeneous category for whom the relationship to active conventional tobacco use is ambiguous.

***Alcohol use outcomes.*** Heavy Drinking was measured with the Alcohol Use Disorders Identification Test Consumption (Audit-C) (Bradley et al., 2007). An Audit-C score greater than 4 was used as cutoff for heavy drinking in men, and 3 for women (Khadjesari et al., 2017). We also examined regular binge drinking defined as having six or more drinks on one occasion on a weekly basis.

***Cannabis use outcomes.*** Finally, we analyzed two variables associated with cannabis use. To obtain the variables, we examined answers to the Cannabis Abuse Screening Test (CAST) scale. This scale assessed cannabis use in the 12 months preceding the survey interviews. The first variable identified users with a high risk of cannabis dependence (i.e. with a CAST score greater than or equal to 7 out of 24) (Spilka and Janssen, 2013), while the second identified the use of cannabis (including hashish, marijuana, weed, a joint or hash) in the 30 days preceding the survey interviews. Questions about cannabis were only asked for participants aged 18 to 64 years old, that is 82% of the unweighted sample (*n* = 20,665).

The analysis of the two outcome variables for alcohol and cannabis allowed us to estimate the association between sexual violence and both the regular and problematic use of these substances.

#### 2.2.3 Covariates

Uniform covariables were used across all substance use outcome models, and were selected based on their documented relevance to substance use behaviors and known associations with sexual violence in the literature. These characteristics were age, country of birth, marital status, education level, employment status, and childhood trauma. The last variable was obtained by summing responses to the following interview questions: “Before age 18, did either of your parents deal with a major health problem or pass away? [1] did you experience serious arguments between your parents or a violent home environment? [2] did your parents divorce or separate? [3]”. We considered that a respondent had a traumatic experience in childhood if they responded “Yes” to at least one of the three questions.

Major depressive episodes were assessed using the standardized Composite International Diagnostic Interview Short Form (CIDI-SF) questionnaire, which measures DSM-IV criteria over the past 12 months. Given that mental health factors such as post-traumatic stress disorder have been shown to partially mediate the association between sexual violence and substance use, no adjustment was made to these variables to avoid collider bias.

### 2.3 Statistical analysis

The distribution of substance use outcomes, lifetime sexual violence experiences, and all covariates are presented for the total sample and by sex. The presented numbers are unweighted, while the percentages are weighted as previously described.

Bivariate analyses were conducted for each substance use variable in both the general population and by sex.

All analyses were stratified by sex to account for differences in drinking patterns between men and women (McHugh et al., 2018). Adjusted odds ratios (ORa) and 95% confidence intervals were obtained for each dependent variable in each model.

For tobacco and alcohol use variables as well as for lifetime sexual violence, the proportion of missing data was 0.4%, at a maximum. Respondents with this missing data were thus excluded from analyses. Around 18.4% of data was missing for cannabis use **outcome** variables, as cannabis questions were only asked to participants aged 18–64 years. These missing data were not imputed to avoid introducing potential bias. To examine whether the association between sexual violence and substance use varied by repetition of sexual violence (testing the hypothesis of cumulative effects), we created a three-level categorical variable: (1) non-victims (no lifetime sexual violence), (2) single assault (SV once), and (3) repeated assault (SV multiple times). Logistic regression models were then fitted with this categorical sexual violence variable as the primary exposure and each substance use outcome as the dependent variable, using non-victims as the reference category.

Data analyses were performed using R version 4.2.3.

### 2.4 Ethical approval

The French Health Barometer surveys were approved by the French Data Protection Authority (Commission Nationale de l'Informatique et des Libertés, CNIL). In accordance with the guidelines of the CNIL, all participants included in this study provided their informed verbal consent to participate, at the start of the telephone interview. All methods were carried out in accordance with relevant guidelines and regulations.

## 3 Results

### 3.1 Descriptive analysis

Respondents had a mean age of 45.9 years (*SD* *=* 16), of which 51.3% were women. The majority of respondents were born in France, lived with a partner and had an education level greater than or equal to the French Baccalaureate (i.e. a high school diploma). More than 50% of respondents were employed and nearly 80% had a household income of over 1500 € per month. Finally, nearly 10% of respondents had symptoms indicative of a moderate to severe depressive episode and more than 40% had traumatic childhood experiences (Table 1).

**Table 1:** Characteristics of French adults aged 18 to 75, Health Barometer survey (weighted %). Mainland France, 2017.

	Unweighted number (% weighted)		
	Overall sample	Women	Men
	*N* = 25,319	12,986 (51.3)	12,333 (48.7)
Age **			
*Weighted average*	45.9 (15.8)	46.2 (15.4)	45.5 (16.3)
*(Standard deviation)*			
*18 to 24*	2846 (11.2)	1410 (10.9)	1435 (11.6)
*25 to 34*	4337 (17.1)	2216 (17.1)	2122 (17.2)
*35 to 44*	4671 (18.5)	2367 (18.2)	2304 (18.7)
*45 to 54*	4925 (19.5)	2502 (19.3)	2423 (19.7)
*55 to 64*	4613 (18.2)	2400 (18.5)	2213 (17.9)
*65 to 75*	3927 (15.5)	2091(16.1)	1836 (14.9)
	
**Nationality**			
*French by birth*	22,426 (88.6)	11,519 (88.7)	10,907 (88.4)
*Non-French*	2893 (11.4)	1467 (11.3)	1426 (11.6)
	
**Living with a partner**			
*No*	9186 (36.3)	4833 (37.2)	4353 (35.3)
*Yes*	16,133 (62.7)	8153 (62.8)	7980 (64.7)
	
Education level *			
*<High school diploma (< Bac)*	12,306 (48.7)	6186 (47.8)	6120 (49.7)
*At least high school diploma (≥ Bac)*	12,952 (51.3)	6768(52.2)	6185 (50.3)
	
Work status **			
*Employed*	14,106 (55.7)	6801 (52.4)	7305 (59.2)
*Student*	1809 (7.2)	928.7 (7.2)	881 (7.1)
*Unemployed & other inactivity*	4162 (16.4)	2533 (19.5)	1628 (13.2)
*Retired*	5242 (20.7)	2723 (21.0)	2519 (20.4)
	
Monthly income ≥ 1500 € **			
*No*	5608 (22.7)	3188 (25.2)	2420 (20.1)
*Yes*	19,056 (77.3)	**9458 (74.8)**	**9598 (79.9)**
Depressive episode **			
*None*	22,781 (90.2)	11,263 (87.0)	11,517 (93.7)
*Light*	115 (0.5)	78 (0.6)	38 (0.3)
*Moderate*	1357 (5.4)	911 (7.0)	446 (3.6)
*Severe*	992 (3.9)	694 (5.4)	298 (2.4)
	
Traumatic childhood event **			
*No*	14,096 (55.7)	6867 (52.9)	7229 (58.6)
*Yes*	11,223 (44.3)	6119 (47.1)	5104 (41.4)

*Note.* AUDIT-C: Alcohol Use Disorders Identification Test Consumption. CAST: Cannabis Abuse Screening Test. *P*-values of the average and frequency comparison tests between men and women: ** p-value<0.0001 * p-value<0.001.

#### 3.1.1 Lifetime sexual violence experiences and substance use

Among the 25,319 respondents, 1468 individuals, or 5.8% of the sample reported experiencing sexual violence in their lifetime (72 respondents refused to answer and 17 others said they did not know). Of these respondents, sexual violence was nearly five times more prevalent in women (9.5%) than in men (2%). Nearly two thirds of respondents (64.0%) had experienced sexual violence more than once and the average age at first experience (or the first assault) was 13.4 years (*SD* = 8). Of respondents who had experienced sexual violence, 65% first experienced sexual violence before the age of 15 (62.9% for women and 75.9% for men).

As shown in Table 2, over 26.9% of respondents reported smoking every day, over 15% reported heavy drinking, and nearly 4.5% reported having at least 6 drinks on the same occasion once a week. Finally, more than 6.4% of respondents reported using cannabis in the last 30 days and more than 25% of regular users had a high cannabis dependence risk as per their CAST score. Analysis by sex showed significant differences, indicating that women are generally less likely to use tobacco, alcohol or cannabis than men (Table 2).

**Table 2:** : Distribution of lifetime sexual violence experiences and substance use outcomes among French adults aged 18 to 75, Health Barometer survey, Mainland France, 2017.

	Unweighted number (% weighted)		
	Overall sample N = 25,319	Women 12,986 (51.3)	Men 12,333 (48.7)
Sexual violence **			
*No*	23,743 (94.2)	**11,688 (90.5)**	**12,055 (98.0)**
*Yes*	1468 (5.8)	**1227 (9.5)**	**241 (2.0)**
	
Age at (first time) sexual violence **			
*Weighted average (Standard deviation)*	*13.4 (7.8)*	***13.8 (8.1)***	***11.5 (5.5)***
	
Repeated sexual violence			
*Yes*	938 (64.0)	793 (64.7)	145 (60.2)
*No*	502 (34.2)	408 (33.3)	94 (38.0)
*Doesn't know or refuses to answer*	27 (1.8)	24 (2.0)	2 (0.9)
	
Daily tobacco use **			
*No*	18,472 (73.1)	**9831 (75.8)**	**8642 (70.2)**
*Yes*	6811 (26.9)	**3139 (24.2)**	**3672 (29.8)**
	
Number of cigarettes consumed daily **			
*Weighted average (Standard deviation)*	*3.6 (7.7)*	*3.0 (6.5)*	*4.3 (8.9)*

*Heavy drinking and/or active alcohol abuse or dependence (AUDIT-C ≥ 3 for women and ≥* 4 for men) **			
*No*	21,504 (84.9)	**11,704 (90.1)**	**9801 (79.5)**
*Yes*	3815 (15.1)	**1283 (9.9)**	**2532 (20.5)**

Six or more drinks on one occasion once a week (Binge Drinking Weekly) **			
*No*	24,148 (95.5)	**12,768 (98.4)**	**11,380 (92.4)**
*Yes*	1152 (4.5)	**212 (1.6)**	**940 (7.6)**
	
Cannabis use in the past 30 days **			
*No*	20,007 (93.6)	**10,503 (96.4)**	**9504 (90.6)**
*Yes*	1374 (6.4)	**388 (3.6)**	**986 (9.4)**
	
Cannabis dependence risk (CAST) *			
*No risk or low risk*	1756 (74.7)	**622 (81.1)**	**1133 (71.6)**
*High risk (CAST* ***≥*** *7)*	594 (25.3)	**145 (18.9)**	**449 (28.4)**

*Note.* P-values of the average and frequency comparison tests between men and women: ** *p* < 0.0001 * *p* < 0.001.

### 3.2 Adjusted analyses

**Fig. 1:**
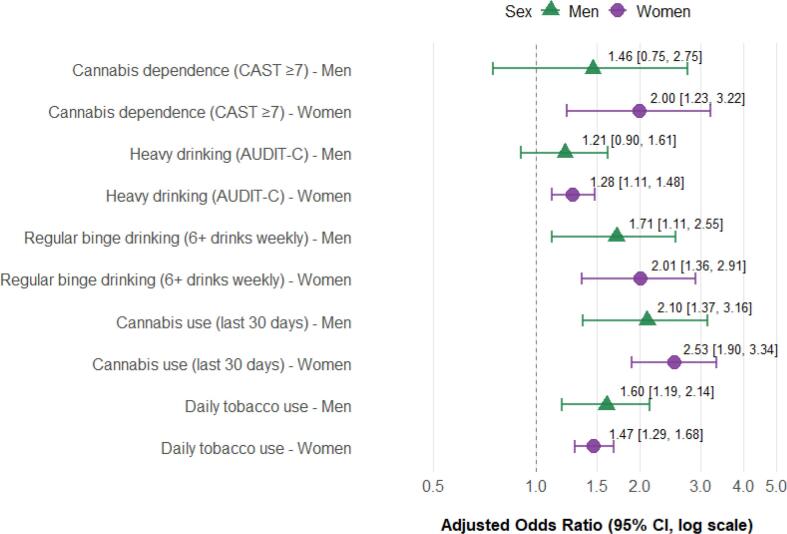
Adjusted association between the experience of lifetime sexual violence and substance use outcomes. Data from the French Health Barometer survey 2017 (French adults aged 18 to 75, Mainland France, 2017). Notes: All models adjusted for age, country of birth, marital status, education level, employment status, and childhood trauma. Reference category: no lifetime sexual violence. Cannabis outcomes restricted to adults aged 18 to 64 years.

Multivariable analyses show, after adjustment, that experiencing sexual violence across a lifetime is associated with substance use in both the total sample and the sample stratified by sex (Fig. 1). We will only be discussing the results of the analysis for the stratified analyses. Results for the total sample and for each model are available in Appendices A to E.

**Tobacco use**. First, we found that the association between lifetime experiences of sexual violence and daily consumption of tobacco is comparable between men (aOR = 1.60, 1.19, 2.14) and women (aOR = 1.47, 1.29, 1.68).

**Alcohol use.** Victims of sexual violence were also more likely to be heavy drinkers in adjusted analyses (aOR = 1.21, 0.90, 1.61 for men / aOR = 1.28, 1.11, 1.48 for women), and regular binge drinkers (aOR = 1.71, 1.11, 2.55 for men / aOR = 2.01, 1.36, 2.91 for women).

**Cannabis use**. Finally, there appears to be a strong association between experiences of sexual violence and cannabis use in the last month (men aOR = 2.10, 1.37, 3.16; women aOR = 2.53, 1.90, 3.34). This association is again stronger for women than for men. For cannabis use at high risk for dependence, the association is strong for women (aOR = 2.00, 1.23, 3.22) but is not found in men (aOR = 1.46, 0.75, 2.75).

#### 3.2.1 Repeated sexual violence

Both single and repeated sexual violence were associated with increased substance use across most outcomes (Supplementary Table S1). However, the pattern of associations was inconsistent across outcomes and by sex, with overlapping confidence intervals.

## 4 Discussion

This analysis, carried out on a representative sample of the French population, shows that people who have experienced sexual violence in their lifetime have an increased risk of using psychoactive substances such as tobacco, alcohol and cannabis. Our results align with previous research on the subject.

Several studies have investigated the association between sexual violence and substance use (Fletcher, 2021). This link may be, at least partly, explained by the intense stress and onset of negative emotions caused by sexual violence. In response to a traumatic sexual violence, victims/survivors often seek to self-medicate to avoid and reduce difficult, negative emotions (Ullman et al., 2005). These often-unsuccessful attempts to disassociate further promote substance use (Holahan et al., 2015; Smith et al., 2014), which in turn negatively affect health, and quality of life (Degenhardt et al., 2018; Rooke et al., 2013).

To better understand the association between sexual violence and substance use, we must consider the impact of other factors as well. While it's important to consider individual characteristics such as mental health pre-assault, biological factors, personality, etc., circumstances of sexual violence also play important role. Research shows that the effects of sexual violence are cumulative, meaning that people who have experienced sexual violence more than once in their life are more likely to use substances (Cisler et al., 2011; Hedtke et al., 2008; Walsh et al., 2014). Our findings offer partial support for this hypothesis: both single and repeated sexual violence were associated with increased substance use risk, though the pattern was inconsistent across outcomes and by sex, and no clear dose-response relationship was demonstrated. The heterogeneity of sexual violence types captured in this study (ranging from unwanted touching to rape) may have contributed to the inconsistent pattern observed across outcomes. Future studies should examine whether the nature and severity of sexual violence, in addition to its repetition, modulates the risk of substance use. Similarly, research shows that the long-term consequences of sexual violence are more numerous when sexual violence took place during childhood, like 65% of the people in our sample (WHO, 2007). The type of sexual violence and the degree of violence also play important roles in the incidence of substance dependence (Campbell et al., 2009; Kendler et al., 2000).

Finally, the impact of sexual violence on the victims/survivor's health can depend on a much wider range of factors, as demonstrated by Rebecca Campbell's ecological model (Campbell et al., 2009). Social reactions, for example, play a determining role in a person's mental health, self-esteem and tendency to self-blame. This may partially explain the differences in substance use outcomes between men and women who had sexual violence experiences in our results.

We acknowledge that this study has several limitations. First, the questionnaire does not allow for the confirmation that respondents began using substances *after* experiencing sexual violence, thus limiting the interpretation of our results. However, our findings on age at sexual violence exposure (mean 13.4 years) indicate that sexual violence occurred at a very young age for respondents in our sample.

Furthermore, the proportions of men and women reporting their sexual violence experiences are most likely underestimated. It is undoubtedly very difficult to talk about such events and it may even lead to dissociative amnesia (Staniloiu and Markowitsch, 2014). However, it should be noted that according to the 2005 Health Barometer, 4.1% of women and 0.6% of men reported experiencing sexual violence in their lifetime (Léon and Lamboy, 2006). Apart from the fact that the survey question was different (the Health Barometer 2005 asked: “In your life, have you been subjected to forced sexual intercourse?”), we can assume that this difference is an outcome of a greater willingness to report sexual violence in a scientific survey, rather than a major increase in the incidence of sexual violence (Bajos et al., 2008). This is a notable development that will perhaps be amplified by the social movements which destigmatized sexual violence subsequent to the collection of our data, but it does not diminish the under-reporting of sexual violence experiences present in our analysis. Moreover, due to the high prevalence of poly-victimization within our sample, disentangling the independent effects of a single incident of sexual violence was not feasible. Furthermore, it should be noted that alcohol and cannabis variables were likely also under-reported in our sample (Le et al., 2022; Stockwell et al., 2016).

The analysis of behavioral differences between men and women in our sample may be underpowered, as suggested by the presented confidence intervals. Only 223 men declared having experienced sexual violence. Finally, this is a cross-sectional study, which does not allow for causality to be determined, and the data is relatively dated since it dates from 2017. To date, it is the last Health Barometer survey that included data on sexual violence in France.

However, this analysis has the advantage of being based on data from a large sample, representative of the general population, making it easier to generalize our results to the entire French adult population. Additionally, this study investigated three distinct psychoactive substances, including different degrees of alcohol and cannabis use. Finally, the multi-thematic nature of the Health Barometer data gives our results a certain robustness. It allowed us to adjust for the impact of a wide range of factors on the association between sexual violence and substance use, and to better take into account the social, demographic and economic diversity of the population.

## 5 Conclusions

The current study presents a significant association between sexual violence experiences and substance use in the general population. It supports the need for adapted and integrated care for victims/survivors, including adequate support and extensive psychological care. Furthermore, based on our results, it may be essential to assess substance abuse patients for a history of sexual violence to guarantee them comprehensive care (medical, psychological, social) that takes into account the multidimensional causes of their dependence.

## Data statement

The Barometre Santé data is the property of Santé Publique France. This data is accessible to public institutions upon formal request. Interested institutions must submit a detailed application outlining the purpose and scope of their intended use. Approval will be granted in accordance with Santé Publique France's data sharing policies and regulations.

## Declaration of generative AI use

DeepL was used to translate portions of the manuscript and improve language. All translated/edited text was reviewed and verified by the authors. Claude (Anthropic) was used to assist in the development of R analysis code. All AI-assisted content was reviewed and verified by the authors.

## CRediT authorship contribution statement

**Noëlla Delmas:** Writing – original draft, Formal analysis, Data curation. **Anne Pasquereau:** Writing – review & editing, Methodology, Data curation, Conceptualization. **Jean-Baptiste Richard:** Writing – review & editing, Validation, Methodology, Data curation. **Viêt Nguyen-Thanh:** Writing – review & editing, Resources, Methodology, Investigation. **Fabienne El-Khoury Lesueur:** Writing – review & editing, Supervision, Methodology, Investigation, Data curation.

## Funding sources

This research did not receive any specific grant from funding agencies in the public, commercial, or not-for-profit sectors.

## Declaration of competing interest

The authors declare that they have no known competing financial interests or personal relationships that could have appeared to influence the work reported in this paper.

## Data Availability

Data will be made available on request.

## References

[bb0005] Bajos N., Bozon M., Csf L. (2008). Les violences sexuelles en France : quand la parole se libère (Sexual violence in France: when speaking out becomes possible.). Population Societes.

[bb0010] Borumandnia N., Khadembashi N., Tabatabaei M., Alavi Majd H. (2020). The prevalence rate of sexual violence worldwide: a trend analysis. BMC Public Health.

[bb0015] Bradley K.A., DeBenedetti A.F., Volk R.J., Williams E.C., Frank D., Kivlahan D.R. (2007). AUDIT-C as a brief screen for alcohol misuse in primary care. Alcohol. Clin. Exp. Res..

[bb0020] Campbell R., Dworkin E., Cabral G. (2009). An ecological model of the impact of sexual assault on women’s mental health. Trauma Violence Abuse.

[bb0025] Choudhary E., Coben J.H., Bossarte R.M. (2008). Gender and time differences in the associations between sexual violence victimization, health outcomes, and risk behaviors. Am. J. Mens Health.

[bb0030] Cisler J.M., Amstadter A.B., Begle A.M., Resnick H.S., Danielson C.K., Saunders B.E., Kilpatrick D.G. (2011). A prospective examination of the relationships between PTSD, exposure to assaultive violence, and cigarette smoking among a National Sample of adolescents. Addict. Behav..

[bb0035] Degenhardt L., Charlson F., Ferrari A., Santomauro D., Erskine H., Mantilla-Herrara A., Whiteford H., Leung J., Naghavi M., Griswold M., Rehm J., Hall W., Sartorius B., Scott J., Vollset S.E., Knudsen A.K., Haro J.M., Patton G., Kopec J., Carvalho Malta D., Topor-Madry R., McGrath J., Haagsma J., Allebeck P., Phillips M., Salomon J., Hay S., Foreman K., Lim S., Mokdad A., Smith M., Gakidou E., Murray C., Vos T. (2018). The global burden of disease attributable to alcohol and drug use in 195 countries and territories, 1990–2016: a systematic analysis for the global burden of disease study 2016. Lancet Psychiatry.

[bb0040] Draucker C.B., Mazurczyk J. (2013). Relationships between childhood sexual abuse and substance use and sexual risk behaviors during adolescence: an integrative review. Nurs. Outlook.

[bb0045] Fletcher K. (2021). A systematic review of the relationship between child sexual abuse and substance use issues. J. Child Sex. Abus..

[bb0050] Hailes H.P., Yu R., Danese A., Fazel S. (2019). Long-term outcomes of childhood sexual abuse: an umbrella review. Lancet Psychiatry.

[bb0055] Halpern S.C., Schuch F.B., Scherer J.N., Sordi A.O., Pachado M., Dalbosco C., Fara L., Pechansky F., Kessler F., Von Diemen L. (2018). Child maltreatment and illicit substance abuse: a systematic review and Meta-analysis of longitudinal studies. Child Abuse Rev..

[bb0060] Hedtke K.A., Ruggiero K.J., Fitzgerald M.M., Zinzow H.M., Saunders B.E., Resnick H.S., Kilpatrick D.G. (2008). A longitudinal investigation of interpersonal violence in relation to mental health and substance use. J. Consult. Clin. Psychol..

[bb0065] Holahan C.J., Moos R.H., Holahan C.K., Cronkite R.C., Randall P.K. (2015). Drinking to cope, emotional distress and alcohol use and abuse: a ten-year model. J. Stud. Alcohol.

[bb0070] Kendler K.S., Bulik C.M., Silberg J., Hettema J.M., Myers J., Prescott C.A. (2000). Childhood sexual abuse and adult psychiatric and substance use disorders in women: an epidemiological and Cotwin control analysis. Arch. Gen. Psychiatry.

[bb0075] Khadjesari Z., White I.R., McCambridge J., Marston L., Wallace P., Godfrey C., Murray E. (2017). Validation of the AUDIT-C in adults seeking help with their drinking online. Addict. Sci. Clin. Pract..

[bb0080] Kish L. (1949). A procedure for objective respondent selection within the household. J. Am. Stat. Assoc..

[bb0085] Le A., Han B.H., Palamar J.J. (2022). Underreporting of past-year cannabis use on a national survey by people who smoke blunts. Subst. Abus..

[bb0090] Léon, C., Lamboy, B., 2006. Les actes de violence physique, in: Baromètre Santé 2005, Premiers Résultats, Baromètres Santé (Physical violence, in: Health Barometer 2005, First Results.). pp. 77–84.

[bb0095] Mandavia A., Robinson G.G.N., Bradley B., Ressler K.J., Powers A. (2016). Exposure to childhood abuse and later substance use: indirect effects of emotion dysregulation and exposure to trauma. J. Trauma. Stress.

[bb0100] McHugh, R.K., Votaw, V.R., Sugarman, D.E., Greenfield, S.F., 2018. Sex and gender differences in substance use disorders. Clin. Psychol. Rev., Gender and Mental Health 66, 12–23. Doi: 10.1016/j.cpr.2017.10.012. PMC5945349 29174306

[bb0105] Piolanti A., Schmid I.E., Fiderer F.J., Ward C.L., Stöckl H., Foran H.M. (2025). Global prevalence of sexual violence against children: a systematic review and Meta-analysis. JAMA Pediatr..

[bb0115] Richard, J.B., Andler, R., Guignard, R., Cogordan, C., Léon, C., Robert, M., 2018. Objectifs, contexte de mise en place et protocole du Baromètre santé 2017 (Objectives, implementation context and protocol of the 2017 Health Barometer.). Saint-Maurice: (Santé publique France 10).

[bb0120] Richard J.-B., Andler R., Gautier A., Guignard R., Leon C., Beck F. (2017). Effects of using an overlapping dual-frame design on estimates of health behaviors: a French general population telephone survey. Journal of Survey Statistics and Methodology.

[bb0125] Rooke S.E., Norberg M.M., Copeland J., Swift W. (2013). Health outcomes associated with long-term regular cannabis and tobacco smoking. Addict. Behav..

[bb0130] Smith K.Z., Smith P.H., Grekin E.R. (2014). Childhood sexual abuse, distress, and alcohol-related problems: moderation by drinking to cope. Psychol. Addict. Behav..

[bb0135] Spilka, S., Janssen, E., 2013. Détection des usages problématiques de cannabis : le cannabis abuse screening test (CAST) (Detection of problematic cannabis use: the Cannabis Abuse Screening Test (CAST).).

[bb0140] Staniloiu A., Markowitsch H.J. (2014). Dissociative amnesia. The lancet. Psychiatry.

[bb0145] Stockwell T., Zhao J., Greenfield T., Li J., Livingston M., Meng Y. (2016). Estimating under- and over-reporting of drinking in national surveys of alcohol consumption: identification of consistent biases across four English-speaking countries. Addiction.

[bb0150] Ullman S.E., Filipas H.H., Townsend S.M., Starzynski L.L. (2005). Trauma exposure, posttraumatic stress disorder and problem drinking in sexual assault survivors. J. Stud. Alcohol.

[bb0160] Walsh K., Resnick H.S., Danielson C.K., McCauley J.L., Saunders B.E., Kilpatrick D.G. (2014). Patterns of drug and alcohol use associated with lifetime sexual revictimization and current posttraumatic stress disorder among three national samples of adolescent, college, and household-residing women. Addict. Behav..

[bb0165] WHO (2007).

[bb0170] World Health Organization (2002). World Report on Violence and Health.

