# Supplementary material for: Lifetime sexual violence and tobacco, alcohol, and cannabis use among French adults: A national survey
Source: Prev Med Rep. 2026 Jun 17;68:103547. doi: 10.1016/j.pmedr.2026.103547 (PMC13311292; doi:10.1016/j.pmedr.2026.103547)
Supplement: Supplementary material 1 [file mmc1.docx]

Supplementary Material. Adjusted associations between lifetime sexual violence and substance use outcomes in the total sample and stratified by sex, Health Barometer survey, Mainland France, 2017

## Appendix A. Adjusted associations between lifetime sexual violence and daily tobacco use among French adults aged 18 to 75, Health Barometer survey, Mainland France, 2017.

| **Outcome** | **Group** | **aOR** | **95% CI** |
| --- | --- | --- | --- |
| Daily tobacco use | Total sample | 1.35 | 1.20, 1.52 |
| Daily tobacco use | Women | 1.47 | 1.29, 1.68 |
| Daily tobacco use | Men | 1.60 | 1.19, 2.14 |

aOR: adjusted odds ratio. CI: confidence interval. All models adjusted for age, country of birth, marital status, education level, employment status, and childhood trauma. Reference category: no lifetime sexual violence.

## Appendix B. Adjusted associations between lifetime sexual violence and cannabis use in the past 30 days among French adults aged 18 to 64, Health Barometer survey, Mainland France, 2017.

| **Outcome** | **Group** | **aOR** | **95% CI** |
| --- | --- | --- | --- |
| Cannabis use (past 30 days) | Total sample | 1.49 | 1.19, 1.84 |
| Cannabis use (past 30 days) | Women | 2.53 | 1.90, 3.34 |
| Cannabis use (past 30 days) | Men | 2.10 | 1.37, 3.16 |

aOR: adjusted odds ratio. CI: confidence interval. All models adjusted for age, country of birth, marital status, education level, employment status, and childhood trauma. Reference category: no lifetime sexual violence.

## Appendix C. Adjusted associations between lifetime sexual violence and regular binge drinking among French adults aged 18 to 75, Health Barometer survey, Mainland France, 2017.

| **Outcome** | **Group** | **aOR** | **95% CI** |
| --- | --- | --- | --- |
| Regular binge drinking (6+ drinks weekly) | Total sample | 0.97 | 0.74, 1.25 |
| Regular binge drinking (6+ drinks weekly) | Women | 2.01 | 1.36, 2.91 |
| Regular binge drinking (6+ drinks weekly) | Men | 1.71 | 1.11, 2.55 |

aOR: adjusted odds ratio. CI: confidence interval. All models adjusted for age, country of birth, marital status, education level, employment status, and childhood trauma. Reference category: no lifetime sexual violence.

## Appendix D. Adjusted associations between lifetime sexual violence and heavy drinking among French adults aged 18 to 75, Health Barometer survey, Mainland France, 2017.

| **Outcome** | **Group** | **aOR** | **95% CI** |
| --- | --- | --- | --- |
| Heavy drinking (Alcohol Use Disorders Identification Test Consumption) | Total sample | 1.04 | 0.92, 1.17 |
| Heavy drinking (Alcohol Use Disorders Identification Test Consumption) | Women | 1.28 | 1.11, 1.48 |
| Heavy drinking (Alcohol Use Disorders Identification Test Consumption) | Men | 1.21 | 0.90, 1.61 |

aOR: adjusted odds ratio. CI: confidence interval. All models adjusted for age, country of birth, marital status, education level, employment status, and childhood trauma. Reference category: no lifetime sexual violence.

## Appendix E. Adjusted associations between lifetime sexual violence and cannabis dependence among French adults aged 18 to 64, Health Barometer survey, Mainland France, 2017.

| **Outcome** | **Group** | **aOR** | **95% CI** |
| --- | --- | --- | --- |
| Cannabis dependence (Cannabis Abuse Screening Test score 7 or above) | Total sample | 1.34 | 0.94, 1.89 |
| Cannabis dependence (Cannabis Abuse Screening Test score 7 or above) | Women | 2.00 | 1.23, 3.22 |
| Cannabis dependence (Cannabis Abuse Screening Test score 7 or above) | Men | 1.46 | 0.75, 2.75 |

aOR: adjusted odds ratio. CI: confidence interval. All models adjusted for age, country of birth, marital status, education level, employment status, and childhood trauma. Reference category: no lifetime sexual violence. Cannabis outcomes restricted to adults aged 18 to 64 years.
