# Supplementary material for: Lifetime sexual violence and tobacco, alcohol, and cannabis use among French adults: A national survey
Source: Prev Med Rep. 2026 Jun 17;68:103547. doi: 10.1016/j.pmedr.2026.103547 (PMC13311292; doi:10.1016/j.pmedr.2026.103547)
Supplement: Supplementary material 2 [file mmc2.docx]

| Supplementary Table S1: Adjusted Associations between Repetition of Sexual Violence and Substance Use | | | | | |
| --- | --- | --- | --- | --- | --- |
| **Outcome** | | **Single Sexual violence experience (SV)** | | **Repeated SV** | |
| **Outcome** | **Sex** | **Single SV aOR (95% CI)** | Single SV **p** | Repeated SV **aOR (95% CI)** | Repeated SV **p** |
| Binge drinking (6+ drinks weekly) | Men | 2.1 [1.05-3.86] | 0.024 | 1.45 [0.81-2.42] | 0.184 |
| Binge drinking (6+ drinks weekly) | Women | 1.96 [1.04-3.4] | 0.026 | 1.84 [1.11-2.91] | 0.013 |
| Cannabis dependence (CAST ≥7) | Men | 1.63 [0.53-4.57] | 0.365 | 1.37 [0.59-2.98] | 0.443 |
| Cannabis dependence (CAST ≥7) | Women | 1.74 [0.84-3.4] | 0.119 | 2.04 [1.12-3.65] | 0.017 |
| Cannabis use (last 30 days) | Men | 2.63 [1.31-5.02] | 0.004 | 1.9 [1.09-3.16] | 0.018 |
| Cannabis use (last 30 days) | Women | 2.19 [1.39-3.34] | <0.001 | 2.64 [1.86-3.69] | <0.001 |
| Daily tobacco use | Men | 1.19 [0.7-1.95] | 0.51 | 1.89 [1.31-2.73] | <0.001 |
| Daily tobacco use | Women | 1.34 [1.07-1.67] | 0.011 | 1.54 [1.3-1.8] | <0.001 |
| Heavy drinking (AUDIT-C) | Men | 1.52 [0.96-2.39] | 0.069 | 1 [0.68-1.46] | 0.99 |
| Heavy drinking (AUDIT-C) | Women | 1.48 [1.18-1.85] | <0.001 | 1.19 [1-1.41] | 0.052 |
| *Note:* |  |  |  |  |  |
| aOR = adjusted odds ratio; CI = 95% confidence interval. All models adjusted for age, country of birth, marital status, household income, education level, employment status, and childhood trauma. Reference category: Non-victims (no lifetime sexual violence). Cannabis outcomes restricted to ages 18-64 years. |  |  |  |  |  |
